# Transcriptomic and Metabolomic Insights into the Inhibitory Mechanisms of Bat Cave Soil Microbial Volatiles Against *Pseudogymnoascus destructans*

**DOI:** 10.3390/microorganisms14071478

**Published:** 2026-07-06

**Authors:** Zihao Huang, Mingqi Shan, Shaopeng Sun, Denghui Wang, Fan Wang, Keping Sun, Zhongle Li, Jiang Feng

**Affiliations:** 1 College of Life Science, Jilin Agricultural University, Changchun 130118, China; mappil3456@163.com (Z.H.);; 2 Key Laboratory of Vegetation Ecology of Education Ministry, Institute of Grassland Science, Northeast Normal University, Changchun 130024, China; 3 Jilin Provincial International Cooperation Key Laboratory for Biological Control of Agricultural Pests, Jilin Agricultural University, Changchun 130118, China; 4 Jilin Provincial Key Laboratory of Animal Resource and Ecological Security, Jilin Agricultural University, Changchun 130117, China

**Keywords:** *Pseudogymnoascus destructans*, white-nose syndrome, volatile organic compounds, multi-omics, antagonistic microorganisms

## Abstract

White-nose syndrome (WNS), caused by the psychrophilic fungus *Pseudogymnoascus destructans*, poses a severe threat to wild bat populations. Caves serve as unique microecosystems. Exploring antagonistic microorganisms and their volatile antifungal compounds within these native environments has emerged as a promising ecological control strategy. In this study, we isolated four antagonistic bacterial strains from bat cave soil that completely inhibit *P. destructans*. Additionally, we identified benzaldehyde (BzH) and 2,5-dimethylpyrazine (2,5-DMP) as their primary antifungal volatile organic compounds (VOCs). Combined physiological, biochemical, and multi-omics analyses revealed that these two VOCs disrupt the structural integrity of the fungal cell wall and membrane. This disruption triggers abnormal energy metabolism and compensatory ATP accumulation, leading to a significant intracellular burst of reactive oxygen species and the impairment of primary antioxidant defenses. This sustained oxidative stress causes irreversible DNA damage, endoplasmic reticulum stress, and basal metabolic dysfunction. Consequently, this cascade induces apoptosis and significantly downregulates the expression of essential virulence genes. In conclusion, this study systematically elucidates the molecular network through which VOCs released by cave soil microorganisms antagonize *P. destructans*. These findings provide a theoretical foundation and candidate intervention molecules for the contactless biocontrol of WNS.

## 1. Introduction

White-nose syndrome (WNS) is an infectious wildlife disease caused by the psychrophilic fungus *Pseudogymnoascus destructans*. *P. destructans* primarily infects the skin tissues of bats during hibernation. This infection triggers frequent abnormal arousals, metabolic disorders, and premature energy depletion, ultimately leading to mass host mortality [1]. Since its emergence in North America in 2006, WNS has killed millions of bats, driving unprecedented population declines [2,3]. Bats are vital ecosystem components, their sharp population decline has profoundly disrupted ecological balances and triggered cascading economic effects [4]. Consequently, developing safe and effective intervention strategies to limit *P. destructans* spread and promote bat population recovery is an urgent priority in wildlife conservation biology.

Current WNS mitigation research largely focuses on chemical reagents or microbial preparations; however, their in situ application in hibernation caves faces spatial limitations and potential toxicological risks [5]. In contrast, volatile-mediated biocontrol strategies leveraging native cave microecosystems offer unique advantages and have garnered widespread attention. Natural cave walls and sediments harbor diverse psychrotolerant microbial communities (e.g., *Pseudomonas* spp., *Bacillus* spp., and *Stenotrophomonas* spp.). These communities exhibit significant in vitro antagonism against *P. destructans*, forming a natural biological barrier against pathogen invasion [6]. The release of volatile organic compounds (VOCs) by these microorganisms is a core mechanism underlying their antifungal efficacy. For instance, *Rhodococcus rhodochrous* DAP96253 effectively inhibits *P. destructans* growth via VOC emission [5,7]. With low molecular weights and high vapor pressures, VOCs diffuse rapidly through the air in complex cave environments. This enables the remote inhibition of pathogenic fungi, effectively circumventing arousal stress in hibernating bats [8]. Previous studies confirm that various VOCs, including 1-pentanol, 2-methyl-1-propanol, and nonanal, exhibit potent antifungal activity against *P. destructans* in vitro and in simulated substrates [8,9].

Recent progress has elucidated the mechanisms by which VOCs antagonize *P. destructans* and other pathogenic fungi. These mechanisms primarily involve disrupting cell wall and membrane integrity, inducing oxidative stress, perturbing energy metabolism, and attenuating pathogenicity [10,11,12,13]. As the primary defense against environmental stress, the fungal cell envelope is the initial target for VOC action. For instance, 1-octanol and (*E*)-2-heptenal inhibit *Aspergillus flavus* growth by interfering with ergosterol synthesis or cell wall remodeling [14,15]. Alongside structural damage, VOCs frequently trigger severe physiological and metabolic dysregulation within these pathogens. For example, ketone volatiles (e.g., 2-undecanone) disrupt the ribosomal stability of *P. destructans* via multitarget pathways [12]. This metabolic stress often precipitates excessive reactive oxygen species accumulation; when the antioxidant defense is overwhelmed, the resulting imbalance causes oxidative DNA damage and triggers apoptosis [16]. Furthermore, certain VOCs directly downregulate the expression of essential virulence factors and metabolites, such as secreted proteases and riboflavin [17]. Despite the promising prospects of VOC-based spatial interventions, their translation into ecological biocontrol agents is hindered by a limited understanding of their underlying mechanisms. Volatiles with distinct molecular structures frequently exhibit significant variations in their specific action targets and subsequent induced cascade pathways when penetrating fungal defenses.

Building upon this foundation, we isolated microorganisms from bat cave soil in northeastern China that completely inhibit *P. destructans*, and identified their primary antifungal VOCs. By integrating physiological, biochemical, transcriptomic, and metabolomic approaches, we systematically evaluated the inhibitory efficacy of these VOCs against *P. destructans* and delineated their interaction networks. Our specific objectives were to: (i) determine the in vitro antifungal activity and dose–response dynamics of the primary volatiles benzaldehyde and 2,5-dimethylpyrazine against *P. destructans*; (ii) assess the disruptive effects of these VOCs on fungal cell envelope integrity, oxidative stress, and energy metabolism; and (iii) elucidate the multi-target molecular mechanisms driving VOC-induced fungal apoptosis via multi-omics analyses. Ultimately, this study systematically elucidates the physiological and molecular mechanisms underlying *P. destructans* inhibition by cave-derived bacterial volatiles, providing candidate intervention molecules and a theoretical framework for WNS biocontrol.

## 2. Materials and Methods

### 2.1. Strains and Chemical Reagents

*P. destructans* strain JH15CN0111a is preserved in Jilin Provincial International Cooperation Key Laboratory for Biological Control of Agricultural Pests at −80 °C [18]. Benzaldehyde (BzH, CAS: 100-52-7, purity 99%) and 2,5-Dimethylpyrazine (2,5-DMP, CAS: 123-32-0, purity 98%) were purchased from Macklin Biochemical Technology Co., Ltd. (Shanghai, China). The superoxide dismutase (SOD) activity assay kit, catalase (CAT) activity assay kit, superoxide anion content assay kit, ATP content assay kit, reactive oxygen species (ROS) assay kit, and hydrogen peroxide (H_2_O_2_) content assay kit were all obtained from Beijing Box Biotechnology Co., Ltd. (Beijing, China). DAPI staining solution and Annexin V-FITC apoptosis detection kit were purchased from Beyotime Biotechnology Co., Ltd. (Shanghai, China). Fungal Fluorescence Stain Kit (CFW Method) was obtained from Solarbio Science and Technology Co., Ltd. (Beijing, China).

### 2.2. Cave Soil Sample Collection and Microbial Isolation

Soil samples for this study were collected in April 2023 from three natural bat caves in northeastern China: Temple Cave (Liaoning Province), Di Cave (Jilin Province), and Gezi Cave (Jilin Province). These caves and the corresponding cave-soil sampling scheme have been described previously [10], but the samples analyzed in the present study were obtained from an independent sampling campaign. To prevent anthropogenic cross contamination of the habitats and ensure operator safety, disposable protective clothing and sterile shoe covers were worn throughout the sampling process. The metal trowels and all related instruments used for sampling were presterilized by autoclaving (121 °C, 20 min). At each sampling site, areas with bat guano accumulation were deliberately avoided, and soil was collected from a depth of 5 to 10 cm below the surface using sterile spatulas. Ten independent sampling points were randomly selected within each cave, yielding a total of 30 samples. Approximately 20 g of soil was collected per sample, placed into a 100 mL sterile sealed glass bottle, transported back to the laboratory on ice within 4 h, and stored at 4 °C for subsequent use.

Isolation and purification were performed according to the method described by Sun et al. [6]. One gram of the homogenized soil sample was weighed and added to 9 mL of sterile water, followed by thorough vortexing. Tenfold serial dilutions were performed, and the 10^−5^ and 10^−6^ dilutions were selected and spread onto beef extract peptone agar and R2A agar plates, respectively, with three biological replicates established for each dilution. The inoculated plates were incubated in a constant temperature and humidity incubator set at 13 °C and 85% relative humidity (RH), and they were continuously observed and recorded at 24, 36, 48, 60, and 72 h. Representative distinct single colonies were picked based on phenotypic characteristics such as colony color, morphology, margin, and elevation. The selected single colonies were successively streak purified three times on the original isolation medium until pure strains were obtained.

### 2.3. Screening and Molecular Identification of Antagonistic Strains

To screen target bacteria exhibiting volatile antifungal activity, we employed a two plate shared space method to evaluate the inhibitory effects of the isolates against *P. destructans*. A 100 μL aliquot of *P. destructans* conidial suspension, adjusted to a concentration of 2 × 10^6^ spores per mL, was evenly spread onto Sabouraud dextrose agar (SDA) plates (90 mm diameter). Simultaneously, a 20 μL volume of the candidate bacterial suspension was inoculated onto Luria Bertani (LB) agar plates (90 mm diameter). The lids of both inoculated plates were removed. They were then placed adjacent to each other within a large sterile Petri dish (200 mm × 30 mm) to create a sealed shared gas space. All setups were incubated continuously for 14 d in a constant temperature incubator set at 13 °C. Three biological replicates were established for the screening assay of each bacterial strain.

Subsequently, the selected highly active antagonistic strains underwent molecular identification via 16S rRNA gene sequence analysis. Bacterial genomic DNA was extracted following the protocol provided with the Tiangen Bacterial Genomic DNA Extraction Kit (Tiangen Biotech, Beijing, China). Polymerase chain reaction amplification was performed using the universal bacterial primers 27F and 1492R [19]. Following sequencing of the amplified products, the resulting reads were spliced and assembled using DNAStar 7.1 software. Finally, the assembled complete sequences were submitted to the NCBI database. The taxonomic status of the antagonistic strains was then determined by performing homology alignments against known sequences in the 16S rRNA database using the BLAST online NCBI BLASTn web server, version BLASTN 2.17.0+.

### 2.4. Collection, Identification, and Antifungal Activity Verification of Volatile Organic Compounds

Headspace solid phase microextraction (HS-SPME) was employed to detect the VOCs released by the four highly active antagonistic strains. The antagonistic bacteria were individually inoculated onto solid LB agar plates and incubated at 13 °C for 14 d. Uninoculated LB plates served as blank controls, and all analytical detections included three biological replicates. The cultured plates were placed into headspace vials and heated at 40 °C for 30 min to promote volatile release. Subsequently, the 50/30 μm divinylbenzene/carboxen/polydimethylsiloxane (DVB/CAR/PDMS) extraction fiber was inserted into the headspace vial and exposed for a 20 min extraction. It was then rapidly transferred to the inlet of a gas chromatography mass spectrometry (Agilent 5975 GC-MS; Agilent Technologies, Santa Clara, CA, USA) system for desorption. Chromatographic separation was achieved using a DB-5MS capillary column (30 m × 0.25 mm × 0.25 μm). Qualitative identification was performed by matching against the NIST/EPA/NIH Mass Spectral Library (NIST 08) mass spectral database utilizing a minimum matching score threshold of 850, and relative abundances were calculated via the peak area normalization method.

Subsequently, pure analytical standards of the compounds exhibiting the highest relative abundances in the VOC profiles of the antagonistic strains were selected to verify their individual antifungal activity against *P. destructans* following a sealed plate fumigation method [10]. Briefly, 100 μL of *P. destructans* conidial suspension, adjusted to 2 × 10^6^ spores per mL, was evenly spread onto SDA plates in 90-mm Petri dishes. The inoculated plates were allowed to air-dry for 10–15 min before VOC exposure. The plates were then inverted so that the lid served as the base. Sterile filter paper discs (6 mm diameter) were placed at the center of the inner surface of the inverted Petri dish lid. BzH (purity 99%) and 2,5-DMP (purity 98%) were used as neat analytical standards without solvent dilution and were directly loaded onto the discs at preset volumes to achieve the target nominal headspace concentrations. The nominal headspace concentration was defined as μL of pure compound per mL of headspace air (μL/mL headspace) and was calculated as follows: C__nom_ = V__VOC_/V__headspace_, where V__VOC_ represents the volume of pure compound loaded onto the disc and V__headspace_ represents the air volume of the sealed plate [11]. The headspace volume was calculated from the plate geometry (90 mm diameter; 20 mm internal height) and the measured agar depth. The dose ranges used for serial dilution were 0.02–0.07 μL/mL headspace for BzH and 0.8–1.3 μL/mL headspace for 2,5-DMP. Blank sterile discs and sterile discs loaded with 95% ethanol were used as negative controls. The 95% ethanol control produced the same result as the blank-disc control, confirming that ethanol alone did not affect *P. destructans* growth under the tested conditions [11]. All treatments included three independent biological replicates.

After VOC loading, the plates were maintained in the inverted position, immediately sealed with Parafilm, and incubated at 13 °C and 85% RH for 14 d. Because the plates were kept inverted, gravity ensured that the discs remained stationary on the lids throughout incubation without the need for adhesives. Upon completion of incubation, the plates were photographed under standardized conditions. Total colony areas were quantified using Ilastik v1.4.1rc2 [20] and ImageJ v1.54f [21], and growth inhibition rates were calculated according to previously published formulas [22]. Target compounds achieving a 100% inhibition rate were selected to further determine their minimum inhibitory concentration (MIC) via serial dilution. Dose response curves were fitted using a four-parameter logistic (4PL) model [23] with the maximum inhibition rate fixed at 100%, thereby yielding the half maximal inhibitory concentration (IC_50_).

### 2.5. Scanning and Transmission Electron Microscopy Observations

*P. destructans* mycelia were treated with 1/2 MIC of BzH and 2,5-DMP under the sealed-plate fumigation conditions described above. The nominal headspace concentrations were 0.035 μL/mL for BzH and 0.65 μL/mL for 2,5-DMP. Untreated mycelia incubated under the same conditions served as controls. All samples were incubated at 13 °C and 85% RH for 14 d before collection. For SEM observation, mycelia were gently collected, washed with sterile PBS, and fixed overnight at 4 °C in 2.5% glutaraldehyde. The fixed samples were rinsed with PBS, dehydrated through a graded ethanol series of 30%, 50%, 70%, 80%, 95%, 100%, and 100%, mounted on aluminum stubs, and gold-sputter-coated following routine SEM preparation procedures. The external morphology of the mycelia was observed using a field-emission scanning electron microscope (Sigma 300; Carl Zeiss Microscopy GmbH, Oberkochen, Germany).

For TEM observation, approximately 1 mm^3^ mycelial blocks were collected and fixed overnight at 4 °C in 2.5% glutaraldehyde. The samples were washed three times with 0.1 M phosphate buffer (PB, pH 7.4) for 15 min each, post-fixed with 1% osmium tetroxide prepared in 0.1 M PB for 7 h at room temperature in the dark, and then washed again three times with PB. Subsequently, the samples were dehydrated through a graded ethanol series of 30%, 50%, 70%, 80%, 95%, 100%, and 100%, transitioned through acetone, infiltrated and embedded in 812 resin, and polymerized at 60 °C for 48 h. Ultrathin sections of 60–80 nm were prepared, mounted on 150-mesh copper grids, stained with 2% uranyl acetate and 2.6% lead citrate for 8 min each, and examined using a transmission electron microscope (HT7800; Hitachi High-Tech Corporation, Tokyo, Japan). Three independent biological replicates were prepared for each treatment, and at least six randomly selected fields were examined per treatment.

### 2.6. Biochemical Assays and Fluorescence Observation of Apoptosis

Mycelia were collected as described in Section 2.5. The mycelia were resuspended in the buffer solutions provided with the respective assay kits at a ratio of 1 g wet weight to 10 mL of extraction buffer. The samples were thoroughly homogenized and disrupted under ice bath conditions, followed by centrifugation at 10,000× *g* for 20 min at 4 °C. The supernatant was collected and stored on ice for subsequent use [24]. The assays for CAT and SOD activities, as well as the determination of superoxide anion, ATP, and H_2_O_2_ contents, were all conducted according to the instructions of the accompanying kits. Upon establishing the reaction systems, absorbance values at the corresponding wavelengths were measured using a UV spectrophotometer and substituted into the integrated equations provided in the manuals to calculate the final results.

The same batch of mycelia under the aforementioned treatment conditions was collected, washed with sterile PBS, and subjected to specific fluorescent probe labeling. Intracellular ROS accumulation was assessed by incubating the samples with a 10 µM 2′,7′-dichlorodihydro-fluorescein diacetate (DCFH-DA) probe at 37 °C for 30 min. Nuclear morphology and DNA damage were evaluated by staining with 4′,6-diamidino-2-phenylindole (DAPI) dye for 5 min. Apoptosis detection was performed utilizing the Annexin V-FITC/PI double staining method. The mycelia were resuspended in 195 µL of Annexin V-FITC binding buffer, followed by the sequential addition of 5 µL of Annexin V conjugated to fluorescein isothiocyanate (Annexin V-FITC) and 15 µL of propidium iodide (PI), and incubated at 4 °C for 20 min. All aforementioned probe incubation processes were strictly conducted in the dark. Nonspecific binding dyes were removed by washing with PBS. The prepared slides were then examined under a confocal laser scanning microscope (TCS SP8; Leica Microsystems CMS GmbH, Wetzlar, Germany) to observe target fluorescence signals and randomly capture images.

### 2.7. Transcriptomics Analysis and RT-qPCR Validation

Treated and untreated mycelia (*n* = 4) were collected under the conditions described in Section 2.5, flash frozen in liquid nitrogen, and stored at −80 °C for subsequent use. Transcriptome sequencing and library construction were performed referencing the methods described by Shi et al. [25]. Total RNA was extracted using the Total RNA Extractor kit (Invitrogen, Waltham, MA, USA), and residual genomic DNA was removed by DNase treatment according to the manufacturer’s protocol. RNA purity and concentration were assessed using a NanoDrop spectrophotometer and a Qubit 2.0 Fluorometer (Invitrogen, Waltham, MA, USA), and RNA integrity was evaluated using an Agilent 2100 Bioanalyzer (Agilent Technologies, Santa Clara, CA, USA). Only high-quality RNA samples with OD_260/280_ values of 1.8–2.2, OD_260/230_ values greater than 1.8, and RNA integrity number (RIN) values greater than 7.0 were used for library construction. Poly(A) mRNA was enriched using Oligo(dT) magnetic beads, and standard mRNA-seq libraries were constructed. After library construction, preliminary quantification was performed using a Qubit 2.0 Fluorometer, and the libraries were diluted to 1.5 ng/μL. Insert size was assessed using an Agilent 2100 Bioanalyzer, and the effective library concentration was accurately quantified by qRT-PCR. Libraries with effective concentrations higher than 1.5 nM and insert sizes within the expected range were subjected to paired-end 150 bp sequencing on an Illumina NovaSeq 6000 platform at Novogene Co., Ltd. (Beijing, China), with an expected sequencing depth of approximately 6 Gb per sample. The raw sequencing data have been submitted to the NCBI SRA database (accession number: PRJNA1450846). 

Quality control of the raw data was performed using fastp software [26] to remove adaptor sequences, low-quality reads, and reads containing excessive undetermined bases. The resulting high-quality clean reads were aligned to the *P. destructans* reference genome ASM164126v1 using HISAT2 v2.0.5 [27] with default parameters. Gene annotation was based on the NCBI annotation file GCF_001641265.1 [28]. Gene-level raw read counts were obtained using featureCounts [29] in paired-end mode by assigning uniquely mapped reads to annotated gene features. Gene expression levels were normalized and expressed as fragments per kilobase of transcript per million mapped reads (FPKM). Differentially expressed genes (DEGs) were identified using the DESeq2 R package v1.20.0 [30], based on raw read counts, and *p*-values were adjusted using the Benjamini–Hochberg method to control the false discovery rate (FDR). The threshold for significant differential expression was set at an FDR < 0.05 and an absolute log_2_ |fold change| > 1. Subsequently, GO functional and KEGG pathway enrichment analyses were conducted using clusterProfiler v3.8.1 software [31], with the significance threshold set at *p* < 0.05.

Total RNA from the same batch used for transcriptome sequencing was taken to synthesize cDNA via reverse transcription using the StarScript Pro All in one RT Mix with gDNA Remover (GeneStar, Nanjing, China). Quantitative reverse transcription PCR (RT qPCR) was performed on a QuantStudio 3 system (Thermo Scientific, Waltham, MA, USA), with the reaction mixtures prepared using the 2× RealStar Fast SYBR qPCR Mix (Low ROX) (GeneStar, Nanjing, China). The thermal cycling program for the 50 µL amplification system was set as follows: initial denaturation at 95 °C for 2 min; followed by 40 cycles of denaturation at 95 °C for 5 s, and annealing and extension at 60 °C for 30 s. The expression levels of nine key target genes were measured using *EFG1* as the internal reference gene (primer sequences are provided in Table S1). The relative expression levels of the target genes were calculated employing the 2^−∆∆Ct^ method.

### 2.8. Metabolomics Analysis

Mycelia from the treatment and control groups (*n* = 7) were collected under the conditions described in Section 2.5. Metabolite extraction and the liquid chromatography tandem mass spectrometry (LC-MS/MS) detection workflow were performed according to the descriptions by Li et al. [32]. Metabolites were extracted using a prechilled methanol/acetonitrile/water solution (2:2:1, *v*/*v*/*v*). Briefly, samples were vortexed, ultrasonicated at low temperature for 30 min, incubated at −20 °C for 10 min, and centrifuged at 14,000× *g* for 20 min at 4 °C. The supernatant was collected and vacuum-dried. For LC-MS/MS analysis, the dried extracts were reconstituted in 100 μL of acetonitrile/water solution (1:1, *v*/*v*), vortexed, and centrifuged at 14,000× *g* for 15 min at 4 °C, and the resulting supernatant was used for analysis. LC-MS/MS analysis was performed using a Vanquish UHPLC system (Thermo Scientific, Waltham, MA, USA) coupled with a Q Exactive HF Orbitrap high-resolution mass spectrometer (Thermo Scientific, Waltham, MA, USA). Chromatographic separation was conducted on a HILIC column at 25 °C with a flow rate of 0.3 mL/min and an injection volume of 2 μL. Mobile phase A consisted of water containing 25 mM ammonium acetate and 25 mM ammonia, and mobile phase B was acetonitrile. The gradient was as follows: 0–1.5 min, 98% B; 1.5–12 min, 98% to 2% B; 12–14 min, 2% B; 14–14.1 min, 2% to 98% B; and 14.1–17 min, 98% B. MS detection was performed in both positive and negative electrospray ionization modes. The full-scan mass range was 80–1200 *m*/*z*, with resolutions of 60,000 for MS1 and 30,000 for MS2. QC samples, prepared by pooling equal aliquots from all samples, were inserted throughout the analytical sequence to monitor system stability, and blank samples were included to identify background signals. No internal standard was used for this untargeted metabolomics analysis.

Raw data were preprocessed using XCMS for peak extraction, retention time correction, and peak alignment [33]. Peaks detected in blank samples were removed or marked as background signals. Missing values were imputed using the K-nearest neighbor (KNN) method. Data normalization was performed using QC-based normalization according to the formula: normalized intensity = raw metabolite intensity/(total metabolite intensity of the sample/total metabolite intensity of QC1). Metabolites were annotated by matching MS/MS spectra against the HMDB, LIPID MAPS, and KEGG databases, and only metabolites meeting Metabolomics Standards Initiative (MSI) level 2 or higher criteria were retained. The normalized data were imported into the MetaboAnalyst 6.0 platform for orthogonal partial least squares discriminant analysis (OPLS-DA). The risk of model overfitting was simultaneously evaluated by incorporating a 200-iteration permutation test [34]. The screening criteria for differentially accumulated metabolites (DEMs) were set at a variable importance in projection (VIP) score > 1 and a *p* value < 0.05. Finally, metabolic pathway enrichment annotation of the screened DEMs was conducted based on the KEGG database, and the relevant data were visualized utilizing the OmicShare tools platform [35].

### 2.9. Integrated Transcriptomics and Metabolomics Analysis

Based on the grouping clustering characteristics of the OPLS-DA model, the corresponding transcriptomic and metabolomic data were extracted for multi-omics joint analysis. Pearson correlation analysis was employed to evaluate the potential associations between DEGs and DEMs. The top 20 key DEGs and DEMs were selected based on ranking by absolute correlation coefficient values. A correlation clustering heatmap was subsequently generated using R software to illustrate the association patterns between specific genes and changes in metabolite abundance.

To further explore the core regulatory networks under the intervention of the active compounds, all DEGs and DEMs identified in this study were jointly mapped to the KEGG database. Joint pathway enrichment analysis was performed using the OmicShare tools platform, thereby identifying the key biological pathways and signal cascades that experienced synergistic perturbation at both the transcriptional and metabolic levels [35].

### 2.10. Statistical Analysis

In this study, the transcriptomic and metabolomic sequencing included four and seven independent biological replicates, respectively, while all remaining experiments incorporated at least three independent biological replicates. All quantitative experimental data are presented as the mean ± standard deviation (SD). Statistical and significance analyses of the data were conducted in SPSS 27.0 (IBM, Armonk, NY, USA). Comparisons between two independent groups of data were performed using Student’s t test; overall differences among multiple groups of data were evaluated via one way analysis of variance (ANOVA), combined with Tukey’s post hoc test for pairwise group comparisons. Differences were considered statistically significant when *p* < 0.05. The generation and formatting of all experimental data charts and graphs were accomplished utilizing Origin 2018 software (OriginLab, Northampton, MA, USA).

## 3. Results

### 3.1. Screening of Antagonistic Soil Microorganisms and the Inhibitory Effects of Active Volatile Compounds

**Figure 1:**
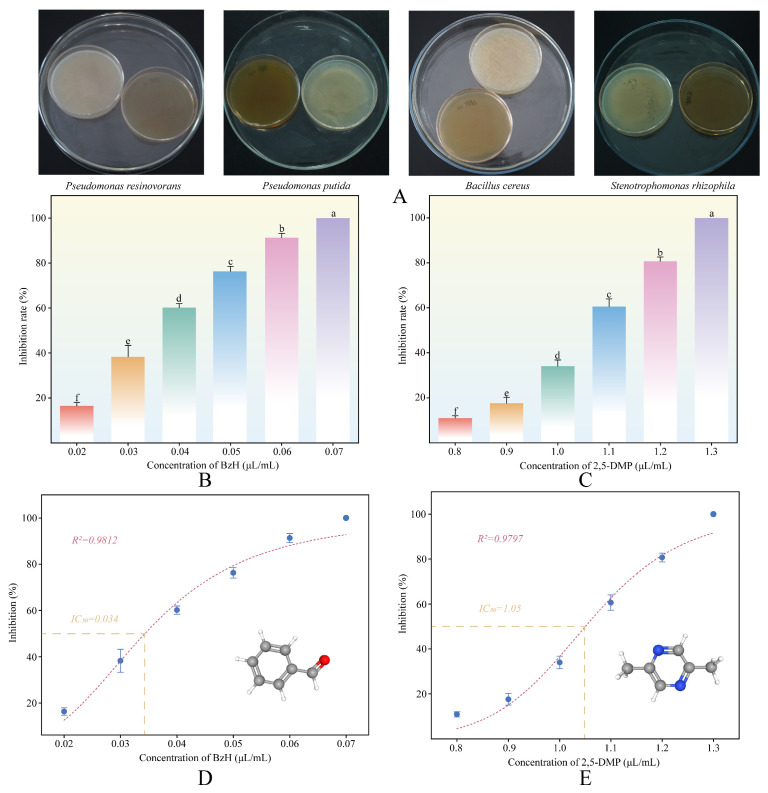
In vitro inhibitory effects of antagonistic bacteria and individual active volatile organic compounds against *P. destructans*. (**A**) Two-plate shared-space co-culture assay showing the spatial inhibition of *P. destructans* by four screened bacterial strains. (**B**,**C**) Inhibition rates of *P. destructans* exposed to different nominal headspace concentrations of BzH (**B**: 0.02–0.07 μL/mL headspace) and 2,5-DMP (**C**: 0.8–1.3 μL/mL headspace) in sealed-plate fumigation assays. The dose unit μL/mL represents μL of pure compound per mL of headspace air, calculated as C__nom_ = V__VOC_/V__headspace_. Data are presented as mean ± SD (*n* = 3). Different lowercase letters above the bars indicate significant differences among concentration groups based on one-way ANOVA followed by Tukey’s post hoc test (*p* < 0.05). (**D**,**E**) Dose–response fitting curves and IC_50_ calculations for BzH (**D**) and 2,5-DMP (**E**), fitted using a four-parameter logistic model with the maximum inhibition rate fixed at 100%. MIC was defined as the lowest nominal headspace concentration that completely inhibited visible mycelial growth after incubation at 13 °C and 85% RH for 14 d. The 3D chemical structures of the respective compounds are shown as insets.

A total of 204 single colonies were isolated from 30 soil samples across the three caves. Several isolates exhibited evident inhibitory activity against *P. destructans* (Table S2). Among these, *Pseudomonas resinovorans*, *Pseudomonas putida*, *Bacillus cereus*, and *Stenotrophomonas rhizophila* demonstrated robust antagonism, completely inhibiting fungal growth (Figure 1A). VOCs profiling of these four highly active strains, combined with plate inhibition assays (Table S3), revealed that two abundant metabolites, benzaldehyde (BzH) and 2,5-dimethylpyrazine (2,5-DMP), completely inhibited *P. destructans* growth.

Dose–response assays further confirmed that the inhibitory activities of both VOCs were concentration-dependent under sealed-plate fumigation conditions (Figure 1B–E). The VOC dose was expressed as the nominal headspace concentration, calculated as μL of pure compound per mL of headspace air as described in Section 2.4. For BzH, the tested concentration range was 0.02–0.07 μL/mL headspace, with an IC_50_ of 0.034 ± 0.001 μL/mL and an MIC of 0.07 μL/mL (R^2^ = 0.9812). For 2,5-DMP, the tested concentration range was 0.8–1.3 μL/mL headspace, with an IC_50_ of 1.05 ± 0.01 μL/mL and an MIC of 1.3 μL/mL (R^2^ = 0.9797). The MIC was defined as the lowest nominal headspace concentration that completely inhibited visible mycelial growth after 14 d of incubation. These results indicate that the individual pure compounds BzH and 2,5-DMP exerted potent concentration-dependent inhibitory effects against *P. destructans* in vitro.

### 3.2. Microscopic Observations of P. destructans Structure Under Active Compound Action

**Figure 2:**
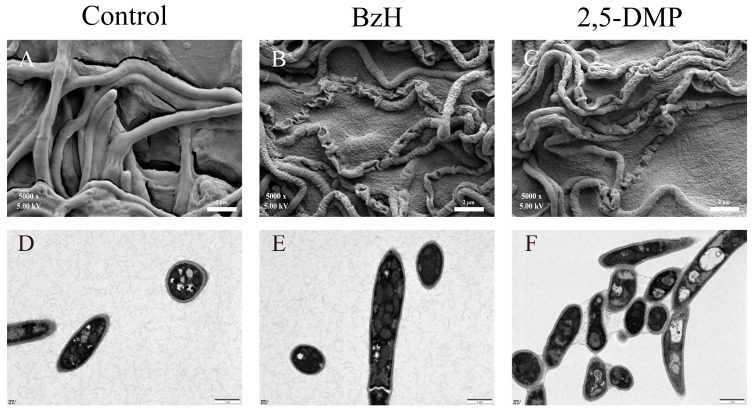
Representative SEM and TEM images of *P. destructans* mycelia after treatment with active compounds. SEM images are shown in panels (**A**–**C**), and TEM images are shown in panels (**D**–**F**). Images from left to right represent the control group (**A**,**D**), BzH treatment group (**B**,**E**), and 2,5-DMP treatment group (**C**,**F**). Magnification: 5000×. Scale bars: 2 μm in (**A**–**C**) and 1 μm in (**D**–**F**).

SEM revealed that control mycelia (Figure 2A) exhibited intact morphology, with smooth surfaces and plump tubular structures. In contrast, BzH- and 2,5-DMP-treated mycelia showed visible surface abnormalities, including wrinkling, tubular collapse, and irregular distortion (Figure 2B,C).

TEM further showed that control mycelial cells (Figure 2D) possessed clear cell envelopes and relatively uniform cytoplasmic density. After VOCs treatment, the mycelia exhibited qualitative ultrastructural alterations. BzH-treated cells (Figure 2E) showed reduced cytoplasmic electron density and less distinct intracellular organization, whereas 2,5-DMP-treated cells (Figure 2F) displayed apparent cellular deformation, irregular cell envelopes, disorganized intracellular contents, and vacuole-like structures. These representative SEM and TEM observations indicate that BzH and 2,5-DMP impair the morphological and ultrastructural integrity of *P. destructans* mycelia.

### 3.3. Effects of Active Compounds on Cell Envelope Integrity and Antioxidant Systems in P. destructans

**Figure 3:**
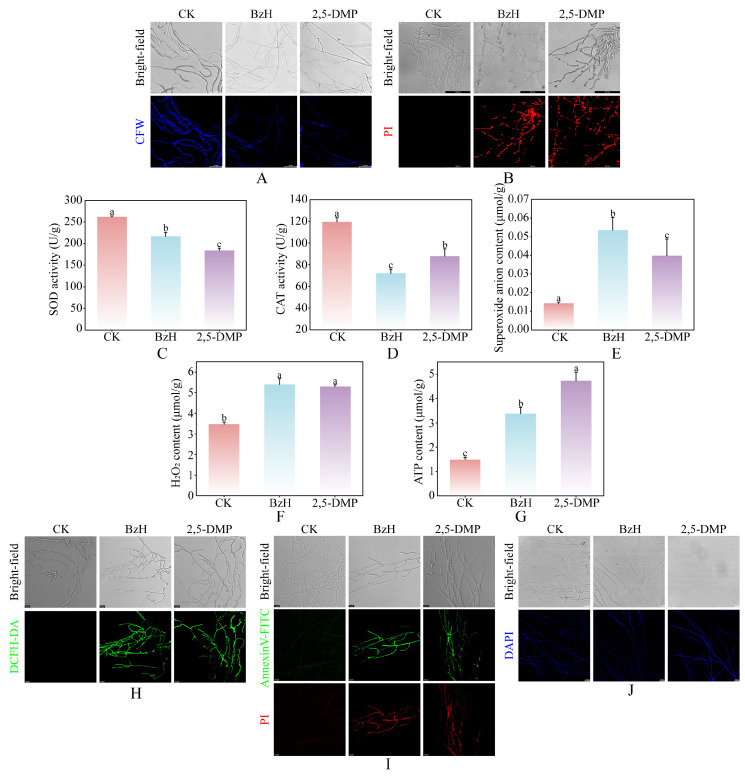
Effects of BzH and 2,5-DMP on cell envelope integrity, oxidative stress, DNA integrity, and apoptosis of *P. destructans*. (**A**) CFW and (**B**) PI staining for cell wall and membrane integrity. (**C**–**G**) Quantitative analysis of SOD activity, CAT activity, superoxide anion content, H_2_O_2_ content, and ATP content. Data are presented as mean ± SD (*n* = 3). Different letters indicate significant differences (*p* < 0.05). (**H**) ROS detection via DCFH-DA. (**I**) Annexin V-FITC/PI apoptosis assay. (**J**) DAPI staining for DNA damage.

CFW staining revealed that control mycelia exhibited continuous and uniform blue fluorescence. Following BzH or 2,5-DMP treatment, the blue fluorescence distribution became uneven, accompanied by localized signal attenuation or complete loss (Figure 3A). PI staining demonstrated an absence of red fluorescence in control mycelia. Conversely, VOC-treated mycelia emitted intense red fluorescence, indicating compromised cell membranes and increased permeability (Figure 3B).

Quantitative biochemical assays revealed a significant decline in antioxidant enzyme activities in the treatment groups compared to the control. Specifically, SOD activity declined to 0.83- and 0.70-fold of the control under BzH and 2,5-DMP treatments, respectively (Figure 3C). Similarly, CAT activity decreased to 0.52- and 0.68-fold, respectively (Figure 3D). Concurrently, ROS significantly accumulated. Superoxide anion contents increased by 3.79- and 2.86-fold under BzH and 2,5-DMP treatments, respectively, while H_2_O_2_ levels rose by 1.55- and 1.52-fold (Figure 3E,F). Furthermore, cellular energy metabolism was significantly disrupted. ATP contents in BzH and 2,5-DMP treated cells increased significantly, reaching 2.26- and 3.17-fold of the control, respectively (Figure 3G). DCFH-DA staining further corroborated this oxidative stress. While control mycelia lacked evident green fluorescence, treated mycelia exhibited intense green signals, indicating massive intracellular ROS accumulation (Figure 3H).

### 3.4. Effects of Active Compounds on Nuclear Morphology and Apoptosis in P. destructans

DAPI staining revealed that nuclei in the control and BzH treated mycelia maintained regular morphologies with uniformly distributed blue fluorescence. Conversely, 2,5-DMP treatment induced nuclear pyknosis, chromatin condensation, and localized fragmentation, indicating DNA damage (Figure 3J). Annexin V-FITC/PI double staining of control mycelia showed a dim background devoid of obvious fluorescence. Following VOC treatment, distinct Annexin V-FITC (green) and PI (red) fluorescence signals were simultaneously detected within the mycelia (Figure 3I). This indicates phosphatidylserine externalization on the cell membrane, signifying early and late apoptosis.

### 3.5. Effects of Active Compounds on the Transcriptome of P. destructans

**Figure 4:**
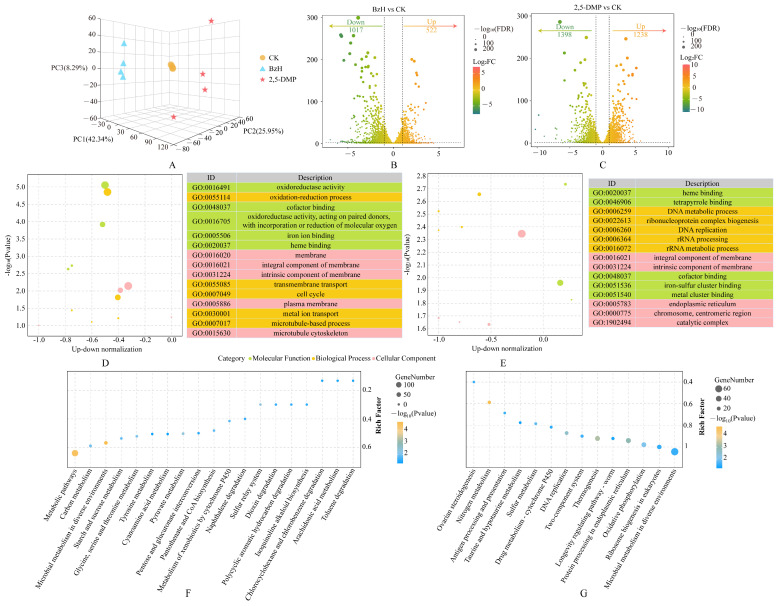
Transcriptomic responses of *P. destructans* to active volatile organic compounds. (**A**) PCA of the transcriptomic data among different groups. (**B**,**C**) Volcano plots showing the distribution of differentially expressed genes (DEGs) in the BzH vs. CK (**B**) and 2,5-DMP vs. CK (**C**) comparison groups. (**D**,**E**) Top 5 enriched GO terms in the biological process, molecular function, and cellular component categories for DEGs under BzH (**D**) and 2,5-DMP (**E**) treatments. (**F**,**G**) Bubble plots displaying the significantly enriched KEGG pathways of DEGs under BzH (**F**) and 2,5-DMP (**G**) treatments.

RNA sequencing generated 31.3 GB of raw data. Quality control indicated that the Q30 values of all samples were at least 95.43%, and the proportion of undetermined bases (N) was 0.01%, confirming that the data quality met the requirements for subsequent analyses (Table S4). PCA revealed distinct spatial separation among the groups (Figure 4A), indicating that VOC treatment altered the overall fungal gene expression patterns. Compared with the control, 1539 DEGs were identified in the BzH treatment group (522 upregulated and 1017 downregulated). Furthermore, 2636 DEGs were identified in the 2,5-DMP treatment group (1238 upregulated and 1398 downregulated) (Figure 4B,C). Additionally, 812 shared DEGs were identified between the two VOC treatment groups.

GO enrichment analysis revealed that DEGs in the BzH treatment group were primarily enriched in oxidation reduction processes and oxidoreductase activity, alongside significant enrichment in cell membrane components (Figure 4D). For the 2,5-DMP treatment group, DEGs were significantly enriched in cell membrane components, DNA replication, metabolic processes, and the endoplasmic reticulum (Figure 4E, Table S5). KEGG pathway analysis demonstrated that the BzH treatment group exhibited significant enrichment in microbial metabolism (ko01120), carbon metabolism (ko01200), pyruvate metabolism (ko00620), and various amino acid metabolic pathways (Figure 4F). Conversely, the 2,5-DMP treatment group showed significant enrichment in nitrogen metabolism (ko00910), protein processing in the endoplasmic reticulum (ko04141), DNA replication (ko03030), and oxidative phosphorylation (ko00190) (Figure 4G, Table S6). Analysis of the 812 shared DEGs indicated significant enrichment in core pathways, including microbial metabolism in diverse environments (ko01120), general metabolic pathways (ko01100), and pyruvate metabolism (ko00620) (Table S7). Collectively, these results suggest that VOCs induced widespread perturbation of basal energy metabolism, disrupted cell membrane structures, and hindered genetic information replication and processing in *P. destructans*.

### 3.6. RT-qPCR Validation of DEGs

To verify transcriptome sequencing data accuracy, nine key DEGs were selected for RT-qPCR analysis. The selected genes encompass those involved in cell wall integrity (*DCW1_1*), protein processing in the endoplasmic reticulum (*IRE1*, *CANX*), heat shock proteins *(HSP70*), DNA replication (*POL30*, *MCM6*), oxidative phosphorylation (*COX10*, *COX17*), and virulence factors (*SP3*). The results demonstrated that under BzH and 2,5-DMP treatments, the expression patterns of these representative genes were consistent with the RNA sequencing data (Figure S1), confirming the reliability of the transcriptomic data.

### 3.7. Effects of Active Compounds on the Metabolomic Profile of P. destructans

**Figure 5:**
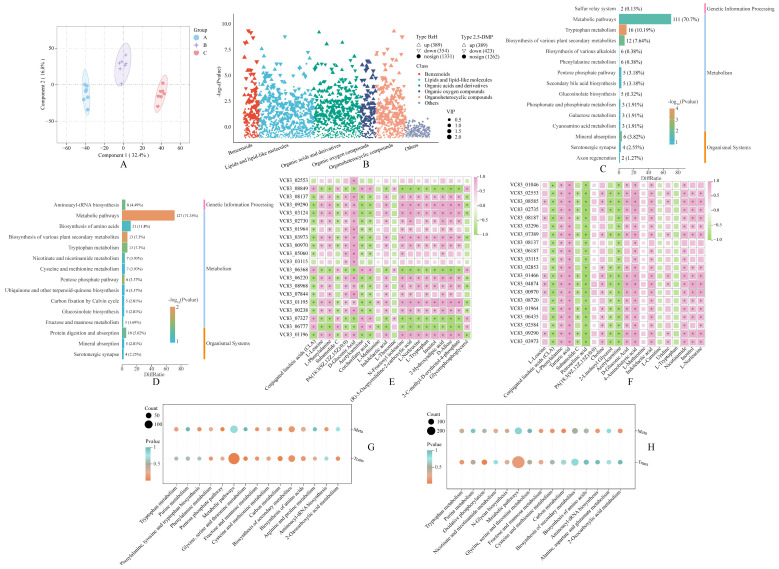
Metabolomic profiling and integrated transcriptomics-metabolomics analysis of *P. destructans* treated with active compounds. (**A**) OPLS-DA score plot of metabolomic samples among the different groups. (**B**) Scatter plot showing the distribution and chemical classification of DEMs under BzH and 2,5-DMP treatments. (**C**,**D**) Top 15 KEGG pathways annotated for DEMs in the BzH (**C**) and 2,5-DMP (**D**) groups. (**E**,**F**) Pearson correlation heatmaps between the top 20 DEGs and DEMs in the BzH (**E**) and 2,5-DMP (**F**) treatment groups. Asterisks (*) denote statistically significant correlations. (**G**,**H**) Bubble plots displaying the KEGG pathways co-enriched by both DEGs and DEMs under BzH (**G**) and 2,5-DMP (**H**) treatments.

OPLS-DA revealed distinct spatial separation between the control and treatment groups (Figure 5A). This indicates that both VOCs induced evident alterations in the overall metabolic profile of *P. destructans*. Subsequently, 2074 metabolites were annotated. Compared with the control, BzH treatment resulted in the differential accumulation of 743 metabolites (389 upregulated and 354 downregulated). For the 2,5-DMP treatment group, 812 DEMs were identified (389 upregulated and 423 downregulated) (Figure 5B). These DEMs predominantly belonged to categories such as benzenoids, lipids, lipidic molecules, and organic acid derivatives.

Further KEGG pathway annotation revealed that under BzH treatment, DEMs were primarily enriched in tryptophan metabolism (ko00380), phosphonate and phosphinate metabolism (ko00440), and the pentose phosphate pathway (ko00030) (Figure 5C). Conversely, in the 2,5-DMP treatment group, DEMs were primarily annotated to the global metabolic network (ko01100), the pentose phosphate pathway (ko00030), tryptophan metabolism (ko00380), and aminoacyl tRNA biosynthesis (ko00970) (Figure 5D, Table S8). These results suggest that VOCs induced dysregulation in the basal metabolism of *P. destructans*, specifically disrupting the homeostasis of core metabolic networks associated with energy supply and amino acid synthesis.

### 3.8. Integrated Transcriptomics and Metabolomics Analysis

A correlation heatmap of the top 20 DEGs and DEMs revealed significant associations between key genes, such as *HSP98* and *HSP78*, and core metabolites, including L-leucine, L-phenylalanine, and L-tryptophan (Figure 5E,F). These results indicate potential associations between VOCs induced transcriptional responses and metabolite abundance changes.

Joint KEGG enrichment analysis demonstrated that under BzH treatment, DEGs and DEMs were mutually enriched in core pathways, including global metabolic pathways (ko01100), secondary metabolite biosynthesis (ko01110), amino acid biosynthesis (ko01230), and carbon metabolism (ko01200) (Figure 5G). In addition to global metabolic pathways and amino acid biosynthesis, the 2,5-DMP treatment group exhibited concurrent enrichment in oxidative phosphorylation (ko00190) and carbon metabolism pathways (Figure 5H). Collectively, these joint analyses indicate that VOCs synergistically disrupted the homeostasis of amino acid synthesis, carbon skeleton metabolism, and energy metabolism in *P. destructans* at both transcriptional and metabolic levels.

## 4. Discussion

The unique oligotrophic, low temperature, and dark habitats of natural bat caves nurture microbial communities with distinct metabolic pathways, representing an important resource repository for discovering antifungal natural products [36]. In this study, we screened multiple antagonistic bacterial strains from cave soils that exhibited a 100% inhibition rate against *P. destructans*. Among them, *Pseudomonas* occurred with highest frequency and demonstrated the most significant antagonistic activity, aligning with previous findings from bat integuments and cave ecosystems in North America and eastern China [37,38,39]. Furthermore, *Bacillus* represents a classical biological control agent; for example, *B. subtilis* 30VD-1 exhibits evident antagonism against *Fusarium* [40]. Notably, *S. rhizophila*, an environmentally friendly biocontrol resource previously reported primarily for its growth promoting and phytopathogen controlling properties (e.g., strain Ep2.2 releases VOCs to inhibit *Botrytis cinerea* growth [41]), further substantiates the excellent antagonistic potential of cave environmental microbes against *P. destructans*.

Given the unique nature of cave habitats, VOC-mediated contactless intervention holds promising application prospects. This constitutes a crucial pathway through which environmental bacteria exert antifungal activity by disrupting core fungal structures [5]. As the primary barrier against environmental stress, the fungal cell envelope is the primary target of VOC attack [42]. CFW staining and SEM observations revealed that VOC treatment severely disrupted the cell wall integrity of *P. destructans* (Figure 2 and Figure 3A). Transcriptomic data further revealed that BzH significantly downregulated the *DCW1_1* (VC83_01650) gene encoding α-1,6-mannosidase, while *DCW1_2* (VC83_02115) and *DCW1_3* (VC83_07145) were significantly downregulated in the 2,5-DMP treatment group. This suggests that VOCs likely inhibited late-stage cell wall processing and remodeling. Previous studies indicate that cave derived VOCs such as nonanal, 2,5-dimethylcyclohexanol, and isovaleric acid disrupt the *P. destructans* cell wall primarily by downregulating chitin synthases [10,11]. These findings supplement the antifungal mechanism of VOCs against *P. destructans* with novel molecular targets. Moreover, cell wall remodeling is a critical mechanism by which *P. destructans* evades host immunity [43]; thus, this damage targeting mannose modifications would not only cause pathogen growth arrest but also drastically attenuate its pathogenicity.

Alongside cell wall damage, TEM and PI staining confirmed irreversible alterations in the cell membrane permeability of *P. destructans* (Figure 2 and Figure 3B). The cell membrane plays a pivotal role in nutrient exchange, stress adaptation, and signal transduction; disrupting its integrity significantly reduces conidial viability and overall survival rates [44]. Joint omics analysis demonstrated that this membrane damage was intimately associated with lipid metabolic network perturbation. At the transcriptional level, VOCs significantly downregulated key ergosterol synthesis genes *ERG1_2* (VC83_06307) and *ERG11* (VC83_07020), aligning with the genetic response of *P. destructans* to phenazine-1-carboxylic acid treatment [16]. At the metabolic level, BzH treatment resulted in significant phosphorylcholine (PCho) downregulation, accompanied by ceramide accumulation. As PCho serves as a precursor for phospholipid synthesis, its obstruction may trigger ceramide mediated apoptosis like programs [45]. In contrast, 2,5-DMP treatment reduced choline and glycerophosphocholine (GPC) contents while simultaneously inducing massive LPC 18:1 production. The reduction in GPC and choline implies membrane lipid turnover mechanism destruction, whereas excessive LPC accumulation may directly compromise lipid bilayer integrity [46].

Cell wall damage and membrane lipid homeostasis disruption typically propagate to energy metabolism systems reliant on membrane structures [47]. Omics results indicated that VOCs did not block the *P. destructans* energy supply but rather induced abnormal energy metabolic stress. Within carbon and pyruvate metabolism pathways, numerous related genes were upregulated, accompanied by declining phosphoenolpyruvate content, indicating accelerated upstream carbon source consumption. In the TCA cycle, although the expression of key enzyme genes (e.g., the SDH family and *FUM1* (VC83_00139)) was upregulated, intermediate metabolite contents like malate and succinate decreased. This suggests increased cyclic metabolic flux and rapid substrate consumption. This abnormal carbon metabolic flux subsequently funneled into the oxidative phosphorylation pathway, causing abnormal electron transport chain (ETC) activation. Following 2,5-DMP treatment specifically, ETC complex genes (e.g., *COX10* and the ATPase complex) were significantly upregulated. Metabolomic NAD^+^ and pyrophosphate accumulation, alongside elevated ATP content in biochemical assays, corroborated highly compensatory respiratory chain activity (Figure 3G). Similar energy metabolism patterns in pathogens facing environmental stress and membrane damage are reported for nonanal treated *P. destructans* and trans-anethole treated *A. flavus* [10,48]. Although short term energy upregulation may be a compensatory cellular response to maintain basic physiological functions like transmembrane potential, abnormal ATP accumulation serves as a prerequisite for inducing apoptosis [49]; biochemical experiments validated this (Figure 3I).

However, sustained high load ETC operation exacerbates electron leakage, leading to massive intracellular ROS accumulation (Figure 3H). For example, 2-phenylethanol inhibits *B. cinerea* growth by inducing excessive ROS accumulation [50]. As oxidative pressure escalated, primary enzymatic antioxidant system function in *P. destructans* was compromised, resulting in significantly declined SOD and CAT activities (Figure 3C,D). Notably, the 2,5-DMP treatment group, which induced higher ETC activity, exhibited a more pronounced decline in SOD activity. Simultaneously, metabolomic data revealed significant pentose phosphate pathway (PPP) enrichment. As the core pathway for intracellular NADPH synthesis, PPP activation is likely a compensatory fungal antioxidant mechanism to maintain redox homeostasis following SOD and CAT enzymatic defense impairment. For instance, when facing host immune cell oxidative stress, *Candida albicans* upregulates PPP genes to sustain intracellular NADPH [51]. High ROS concentrations concurrently promote apoptosis inducing factor release [47]. In summary, VOCs may induce excessive energy metabolism stress by disrupting membrane structures, thereby causing ROS overload and breaching cellular antioxidant defenses, ultimately triggering apoptosis; this likely constitutes the key mechanism underlying their inhibition of *P. destructans* growth.

PPP activation not only provides NADPH but also generates precursors required for nucleotide synthesis. In this study, the purine metabolism pathway and related metabolites were significantly upregulated following 2,5-DMP treatment, suggesting intracellular nucleotide amplification in *P. destructans*. This aligns with the strategy employed by *Candida albicans* to guarantee DNA repair substrate supply via metabolic remodeling in response to host oxidative killing [52]. Excessive ROS readily induce oxidative DNA damage, such as 8-oxoguanine formation [53]. Transcriptomic analysis revealed activation of the base excision repair pathway targeting this damage, evidenced by upregulated *OGG1* (VC83_04837) and *PARP* (VC83_05961) expression. In oxidative stress models of *Saccharomyces cerevisiae*, *OGG1* mediated repair is similarly confirmed as the core mechanism clearing oxidative DNA damage [54]. Concurrently, *P. destructans* exhibited an evident DNA damage response. Core components participating in DNA replication (the MCM complex, PCNA, and the DNA polymerase α subunit) and cell cycle related genes displayed downward trends, indicating that cells might restrict damaged genome replication by arresting cycle progression. However, when sustained oxidative damage exceeds cellular repair capacity, signal transduction undergoes a transition. In the 2,5-DMP treatment group, the core kinase of the cAMP signaling pathway, *PKA* (VC83_05871), was significantly activated. Studies in various fungi (e.g., *S. cerevisiae* and *A. fumigatus*) establish that abnormal PKA pathway overactivity is a key regulatory hub mediating cellular repair abandonment in favor of programmed death [55,56]; biochemical assays confirmed this phenomenon (Figure 3I). In conclusion, VOC-induced ROS accumulation not only induces oxidative DNA damage but likely mediates apoptosis in *P. destructans* by ultimately activating the PKA pathway.

Oxidative stress and carbon metabolism fluctuations may further impact the amino acid metabolic network of *P. destructans*. Metabolomic data indicated that significant carbon skeleton accumulation (e.g., ribose-5-phosphate), alongside chorismate intermediates like prephenate and L-arogenate, elevated aromatic amino acids (including tryptophan and phenylalanine) and various free amino acids. Specifically, notable indole and derivative accumulation in tryptophan metabolism suggests the fungus attempts to alleviate ROS stress by synthesizing secondary metabolites with antioxidant properties [57]. Furthermore, significant aminoacyl tRNA biosynthesis pathway enrichment, coupled with marked upregulation of heat shock protein genes (e.g., *HSP78* (VC83_00970) and *HSP98* (VC83_08137)), indicates intracellular protein turnover system imbalance. Simultaneously, extensive gene expression fluctuations within the endoplasmic reticulum protein processing pathway suggest that high ROS levels induce protein oxidative damage and misfolding, thereby triggering endoplasmic reticulum stress. Similar mechanisms are reported in other fungi; for example, sophorolipids exhibit antifungal activity against *C. albicans* by provoking ROS-mediated endoplasmic reticulum stress [58]. Under energy substrate depletion and structural damage, defensive compensatory stress protein synthesis may be difficult to maintain. This highly energy consuming metabolic remodeling, while failing to effectively repair damaged cells, likely exacerbates the metabolic burden on *P. destructans*, promoting apoptosis.

Beyond disrupting basal physiological metabolism, omics data suggest that VOCs potentially attenuate the pathogenic potential of *P. destructans*. Host colonization and infection by *P. destructans* heavily rely on coordinated specific virulence gene expression [43]. In this study, multiple core virulence pathways were significantly inhibited in the treatment groups (Table S9). These include secreted proteases involved in degrading host tissues (e.g., subtilisin like protease 3 and aspartic type endopeptidases), alongside zinc and calcium ion transport systems competing for microenvironment trace elements, and cell-remodeling-related genes responsible for morphological adaptation and immune evasion [43]. Additionally, widespread perturbation of core molecular chaperone gene expression (e.g., *Hsp70* and *Hsp90*) within the heat shock pathway implies compromised *P. destructans* defense mechanisms against host environmental stress [59]. Metabolically, riboflavin content, a verified characteristic WNS virulence factor, decreased significantly; this substance typically assists the pathogen in resisting oxidative damage during infection and possesses cytotoxicity [60]. The synchronous downregulation of these key infection pathways and virulence metabolites indicates that VOCs, while inducing physiological exhaustion in *P. destructans*, effectively attenuate the basal pathogenicity required for colonizing and destroying host tissues.

Although this study systematically uncovers the multiple antifungal mechanisms of BzH and 2,5-DMP against *P. destructans*, translating these findings into field biocontrol strategies for WNS faces practical challenges. First, the current mechanistic conclusions are primarily based on in vitro controlled systems. Because the sealed-plate fumigation assay used neat analytical standards at nominal headspace concentrations, these exposure levels should be interpreted as controlled mechanistic doses rather than direct estimates of VOC concentrations naturally produced by bacterial cultures or maintained in cave air. Natural cave ecosystems feature complex temperature and humidity fluctuations, airflow exchange, substrate adsorption, and mixed microbial volatile profiles, all of which may alter effective VOC concentrations and action durations [5]. In addition, BzH and 2,5-DMP may coexist under natural conditions rather than acting as isolated compounds. Previous studies have shown that cave-derived VOCs, such as isovaleric acid and ethyl methyl carbonate, can exert synergistic inhibitory effects against *P. destructans* [11], suggesting that interactions among volatile compounds may substantially influence antifungal efficacy. Therefore, although the present study evaluated BzH and 2,5-DMP separately, their combined effects may be additive, synergistic, or even antagonistic depending on their concentration ratios, volatility, and environmental retention. Furthermore, implementing spatial interventions in natural environments requires rigorous evaluation of whether these compounds pose potential toxicity to hibernating wild bats or disrupt the native cave microecological balance. Therefore, subsequent research must incorporate simulated cave systems or in vivo bat infection models. These studies should focus on VOC field safety and controlled release technologies, providing a practical translational blueprint for disease prevention and control in wild bat populations.

## 5. Conclusions

In this study, we screened four antagonistic bacterial strains from cave soil exhibiting complete inhibitory activity against *P. destructans*. We confirmed that their abundant volatiles, BzH and 2,5-DMP, possess potent antifungal efficacy. Mechanistic analyses indicate that these two VOCs primarily disrupt cell envelope structural integrity. Cellular barrier impairment triggers abnormal energy stress, leading to respiratory chain hyperactivity and ATP accumulation. Consequently, this cascade provokes massive reactive oxygen species accumulation and severe antioxidant defense system perturbation. Sustained oxidative stress causes profound DNA damage and endoplasmic reticulum stress, accompanied by severe basal metabolism dysregulation, including amino acid synthesis. Simultaneously, core virulence gene expression and critical host infection metabolite production are significantly downregulated. This multitarget synergistic destruction ultimately induces fungal apoptosis. This study systematically elucidates the physiological and molecular mechanisms by which cave-derived VOCs inhibit *P. destructans*, providing promising candidate molecules and theoretical support for WNS biocontrol.

## Data Availability

The raw transcriptome data has been stored in the NCBI SRA database, accession number: PRJNA1450846. The raw metabolomics data is stored in the Figshare database and can be obtained via https://doi.org/10.6084/m9.figshare.31995825 (accessed on 2 July 2026).

## Supplementary Materials

The following supporting information can be downloaded at: https://www.mdpi.com/article/10.3390/microorganisms14071478/s1, Figure S1. RT-qPCR validation of RNA-seq data. Table S1. Primers used for RT-qPCR. Table S2. Taxonomic identification and antifungal activity of the top 10 microbial strains. Table S3. GC-MS identification of the top five most abundant volatile organic compounds produced by the four highly antagonistic bacterial strains after blank-control comparison. Table S4. Overview of RNA-seq read quality. Table S5. Top 5 enriched GO terms in each primary category for DEGs following BzH and 2,5-DMP treatments. Table S6. Significantly enriched KEGG pathways of DEGs in the BzH and 2,5-DMP treatment groups. Table S7. Significantly enriched KEGG pathways of shared DEGs in *P. destructans* under BzH and 2,5-DMP treatments. Table S8. Top 15 enriched KEGG pathways of DEMs in the BzH and 2,5-DMP treatment groups. Table S9. List of putative virulence factors.

## References

[1] Warnecke L., Turner J.M., Bollinger T.K., Misra V., Cryan P.M., Blehert D.S., Wibbelt G., Willis C.K. (2013). Pathophysiology of white-nose syndrome in bats: A mechanistic model linking wing damage to mortality. Biol. Lett..

[2] Blehert D.S., Hicks A.C., Behr M., Meteyer C.U., Berlowski-Zier B.M., Buckles E.L., Coleman J.T., Darling S.R., Gargas A., Niver R. (2009). Bat white-nose syndrome: An emerging fungal pathogen?. Science.

[3] Hoyt J.R., Kilpatrick A.M., Langwig K.E. (2021). Ecology and impacts of white-nose syndrome on bats. Nat. Rev. Microbiol..

[4] Frank E.G. (2024). The economic impacts of ecosystem disruptions: Costs from substituting biological pest control. Science.

[5] Gabriel K.T., McDonald A.G., Lutsch K.E., Pattavina P.E., Morris K.M., Ferrall E.A., Crow S.A., Cornelison C.T. (2022). Development of a multi-year white-nose syndrome mitigation strategy using antifungal volatile organic compounds. PLoS ONE.

[6] Sun S., Shan M., Huang Z., Lv Y., Wei Z., Shen M., Sun K., Li Z., Feng J. (2025). Screening microbial inhibitors of *Pseudogymnoascus destructans* in Northern China. Microbiol. Spectr..

[7] Cornelison C.T., Keel M.K., Gabriel K.T., Barlament C.K., Tucker T.A., Pierce G.E., Crow S.A. (2014). A preliminary report on the contact-independent antagonism of *Pseudogymnoascus destructans* by *Rhodococcus rhodochrous* strain DAP96253. BMC Microbiol..

[8] Micalizzi E.W., Smith M.L. (2020). Volatile organic compounds kill the white-nose syndrome fungus, *Pseudogymnoascus destructans*, in hibernaculum sediment. Can. J. Microbiol..

[9] Cornelison C.T., Gabriel K.T., Barlament C., Crow S.A. (2014). Inhibition of *Pseudogymnoascus destructans* growth from conidia and mycelial extension by bacterially produced volatile organic compounds. Mycopathologia.

[10] Huang Z., Shan M., Li A., Wang K., Wei Z., Shen M., Lu J., Sun K., Li Z., Feng J. (2025). Mechanisms of volatile organic compounds from bat cave environments against *Pseudogymnoascus destructans* in vitro. Appl. Environ. Microbiol..

[11] Huang Z., Sun S., Li Y., Wei Z., Shen M., Lu J., Sun K., Li Z., Feng J. (2026). Inhibitory and synergistic effects of volatile organic compounds from bat caves against *Pseudogymnoascus destructans* in vitro. mSystems.

[12] Lu Y., Zhu Y., Huang L., Pu Y., Sun X., Feng J., Sun K. (2025). Antifungal mechanism of ketone volatile organic compounds against *Pseudogymnoascus destructans*. Virulence.

[13] Zhao X., Zhou J., Tian R., Liu Y. (2022). Microbial volatile organic compounds: Antifungal mechanisms, applications, and challenges. Front. Microbiol..

[14] Qin Y., Zhang S., Lv Y., Zhai H., Hu Y., Cai J. (2022). The antifungal mechanisms of plant volatile compound 1-octanol against *Aspergillus flavus* growth. Appl. Microbiol. Biotechnol..

[15] Duan W., Zhang S., Lv Y., Zhai H., Wei S., Ma P., Cai J., Hu Y. (2023). Inhibitory effect of (*E*)-2-heptenal on *Aspergillus flavus* growth revealed by metabolomics and biochemical analyses. Appl. Microbiol. Biotechnol..

[16] Huang Z., Sun S., Jin Z., Ji Y., Lu J., Xu T., Sun K., Li Z., Feng J. (2025). Multi-omics analyses reveal the antifungal mechanism of phenazine-1-carboxylic acid against *Pseudogymnoascus destructans*. J. Fungi.

[17] Korn V.L., Pennerman K.K., Padhi S., Bennett J.W. (2021). *Trans*-2-hexenal downregulates several pathogenicity genes of *Pseudogymnoascus destructans*, the causative agent of white-nose syndrome in bats. J. Ind. Microbiol. Biotechnol..

[18] Hoyt J.R., Sun K., Parise K.L., Lu G., Langwig K.E., Jiang T., Yang S., Frick W.F., Kilpatrick A.M., Foster J.T. (2016). Widespread bat white-nose syndrome fungus, northeastern China. Emerg. Infect. Dis..

[19] Frank J.A., Reich C.I., Sharma S., Weisbaum J.S., Wilson B.A., Olsen G.J. (2008). Critical evaluation of two primers commonly used for amplification of bacterial 16S rRNA genes. Appl. Environ. Microbiol..

[20] Berg S., Kutra D., Kroeger T., Straehle C.N., Kausler B.X., Haubold C., Schiegg M., Ales J., Beier T., Rudy M. (2019). ilastik: Interactive machine learning for (bio)image analysis. Nat. Methods.

[21] Schneider C.A., Rasband W.S., Eliceiri K.W. (2012). NIH Image to ImageJ: 25 years of image analysis. Nat. Methods.

[22] Micalizzi E.W., Mack J.N., White G.P., Avis T.J., Smith M.L. (2017). Microbial inhibitors of the fungus *Pseudogymnoascus destructans*, the causal agent of white-nose syndrome in bats. PLoS ONE.

[23] Xun W., Gong B., Liu X., Yang X., Zhou X., Jin L. (2023). Antifungal mechanism of phenazine-1-carboxylic acid against *Pestalotiopsis kenyana*. Int. J. Mol. Sci..

[24] Lv T., Li J., Zhou L., Zhou T., Pritchard H.W., Ren C., Chen J., Yan J., Pei J. (2024). Aging-induced reduction in safflower seed germination via impaired energy metabolism and genetic integrity is partially restored by sucrose and DA-6 treatment. Plants.

[25] Shi Q., Zhang X., Zhang Z., Wang N., Liu H., Zhang R., Sun A., Chen J., Shi X. (2023). Transcriptome sequencing and metabolite analysis reveal the single and combined effects of microplastics and di-(2-ethylhexyl) phthalate on *Peneaus vannamei*. Sci. Total Environ..

[26] Chen S. (2025). fastp 1.0: An ultra-fast all-round tool for FASTQ data quality control and preprocessing. iMeta.

[27] Pertea M., Kim D., Pertea G.M., Leek J.T., Salzberg S.L. (2016). Transcript-level expression analysis of RNA-seq experiments with HISAT, StringTie and Ballgown. Nat. Protoc..

[28] Palmer J.M., Drees K.P., Foster J.T., Lindner D.L. (2018). Extreme sensitivity to ultraviolet light in the fungal pathogen causing white-nose syndrome of bats. Nat. Commun..

[29] Liao Y., Smyth G.K., Shi W. (2014). featureCounts: An efficient general purpose program for assigning sequence reads to genomic features. Bioinformatics.

[30] Liu S., Wang Z., Zhu R., Wang F., Cheng Y., Liu Y. (2021). Three Differential Expression Analysis Methods for RNA Sequencing: Limma, EdgeR, DESeq2. J. Vis. Exp..

[31] Xu S., Hu E., Cai Y., Xie Z., Luo X., Zhan L., Tang W., Wang Q., Liu B., Wang R. (2024). Using clusterProfiler to characterize multiomics data. Nat. Protoc..

[32] Li Y., Zhang S., Lv Y., Zhai H., Cai J., Hu Y. (2022). Linalool, the main volatile constituent from *Zanthoxylum schinifolium* pericarp, prevents growth of *Aspergillus flavus* in post-harvest grains. Food Control.

[33] Smith C.A., Want E.J., O’Maille G., Abagyan R., Siuzdak G. (2006). XCMS: Processing mass spectrometry data for metabolite profiling using nonlinear peak alignment, matching, and identification. Anal. Chem..

[34] Pang Z., Lu Y., Zhou G., Hui F., Xu L., Viau C., Spigelman A.F., MacDonald P.E., Wishart D.S., Li S. (2024). MetaboAnalyst 6.0: Towards a unified platform for metabolomics data processing, analysis and interpretation. Nucleic Acids Res..

[35] Mu H., Chen J., Huang W., Huang G., Deng M., Hong S., Ai P., Gao C., Zhou H. (2024). OmicShare tools: A zero-code interactive online platform for biological data analysis and visualization. iMeta.

[36] Kosznik-Kwasnicka K., Golec P., Jaroszewicz W., Lubomska D., Piechowicz L. (2022). Into the unknown: Microbial communities in caves, their role, and potential use. Microorganisms.

[37] Leng H., Sun X., Pu Y., Huang L., Dai W., Feng J., Sun K. (2026). Role of anti-*Pseudogymnoascus destructans* bacteria in cave ecosystems during bat hibernation in northeast China. Appl. Environ. Microbiol..

[38] Li Z., Li A., Hoyt J.R., Dai W., Leng H., Li Y., Li W., Liu S., Jin L., Sun K. (2022). Activity of bacteria isolated from bats against *Pseudogymnoascus destructans* in China. Microb. Biotechnol..

[39] Hoyt J.R., Cheng T.L., Langwig K.E., Hee M.M., Frick W.F., Kilpatrick A.M. (2015). Bacteria isolated from bats inhibit the growth of *Pseudogymnoascus destructans*, the causative agent of white-nose syndrome. PLoS ONE.

[40] Khan N., Martinez-Hidalgo P., Ice T.A., Maymon M., Humm E.A., Nejat N., Sanders E.R., Kaplan D., Hirsch A.M. (2018). Antifungal activity of *Bacillus* species against *Fusarium* and analysis of the potential mechanisms used in biocontrol. Front. Microbiol..

[41] Raio A., Brilli F., Neri L., Baraldi R., Orlando F., Pugliesi C., Chen X., Baccelli I. (2023). *Stenotrophomonas rhizophila* Ep2.2 inhibits growth of *Botrytis cinerea* through the emission of volatile organic compounds, restricts leaf infection and primes defense genes. Front. Plant Sci..

[42] Usman S., Du C., Qin Q., Odiba A.S., He R., Wang B., Jin C., Fang W. (2022). Phosphomannose isomerase is involved in development, stress responses, and pathogenicity of *Aspergillus flavus*. Microbiol. Spectr..

[43] Reeder S.M., Palmer J.M., Prokkola J.M., Lilley T.M., Reeder D.M., Field K.A. (2017). *Pseudogymnoascus destructans* transcriptome changes during white-nose syndrome infections. Virulence.

[44] He R., Xie J., Hao J., Wang B., Jin C., Fang W. (2026). Identification and characterization of a novel broad-spectrum antifungal compound targeting the *Aspergillus fumigatus* cell wall and cell membrane and inducing oxidative stress. Microbiol. Spectr..

[45] Lagace T.A., Ridgway N.D. (2013). The role of phospholipids in the biological activity and structure of the endoplasmic reticulum. Biochim. Biophys. Acta.

[46] Henry S.A., Kohlwein S.D., Carman G.M. (2012). Metabolism and regulation of glycerolipids in the yeast *Saccharomyces cerevisiae*. Genetics.

[47] OuYang Q., Tao N., Zhang M. (2018). A Damaged oxidative phosphorylation mechanism is involved in the antifungal activity of citral against *Penicillium digitatum*. Front. Microbiol..

[48] Lei J., Li Q., Zhang S., Lv Y., Zhai H., Wei S., Ma P., Hu Y. (2023). Transcriptomic and biochemical analyses revealed antifungal mechanism of *trans*-anethole on *Aspergillus flavus* growth. Appl. Microbiol. Biotechnol..

[49] Zamaraeva M.V., Sabirov R.Z., Maeno E., Ando-Akatsuka Y., Bessonova S.V., Okada Y. (2005). Cells die with increased cytosolic ATP during apoptosis: A bioluminescence study with intracellular luciferase. Cell Death Differ..

[50] Zou X., Wei Y., Jiang S., Xu F., Wang H., Zhan P., Shao X. (2022). ROS stress and cell membrane disruption are the main antifungal mechanisms of 2-phenylethanol against *Botrytis cinerea*. J. Agric. Food Chem..

[51] da Silva Dantas A., Day A., Ikeh M., Kos I., Achan B., Quinn J. (2015). Oxidative stress responses in the human fungal pathogen, *Candida albicans*. Biomolecules.

[52] Thomson G.J., Hernon C., Austriaco N., Shapiro R.S., Belenky P., Bennett R.J. (2019). Metabolism-induced oxidative stress and DNA damage selectively trigger genome instability in polyploid fungal cells. EMBO J..

[53] Chiorcea-Paquim A.M. (2022). 8-oxoguanine and 8-oxodeoxyguanosine biomarkers of oxidative DNA damage: A review on HPLC-ECD determination. Molecules.

[54] Girard P.M., Guibourt N., Boiteux S. (1997). The Ogg1 protein of *Saccharomyces cerevisiae*: A 7,8-dihydro-8-oxoguanine DNA glycosylase/AP lyase whose lysine 241 is a critical residue for catalytic activity. Nucleic Acids Res..

[55] Giannattasio S., Guaragnella N., Zdralevic M., Marra E. (2013). Molecular mechanisms of *Saccharomyces cerevisiae* stress adaptation and programmed cell death in response to acetic acid. Front. Microbiol..

[56] Zhao W., Panepinto J.C., Fortwendel J.R., Fox L., Oliver B.G., Askew D.S., Rhodes J.C. (2006). Deletion of the regulatory subunit of protein kinase A in *Aspergillus fumigatus* alters morphology, sensitivity to oxidative damage, and virulence. Infect. Immun..

[57] Chen Y., Yang X., Zhang L., Wu Q., Li S., Gou J., He J., Zhang K., Li S., Niu X. (2023). Tryptophan-centered metabolic alterations coincides with lipid-mediated fungal response to cold stress. Heliyon.

[58] Haque F., Verma N.K., Alfatah M., Bijlani S., Bhattacharyya M.S. (2019). Sophorolipid exhibits antifungal activity by ROS mediated endoplasmic reticulum stress and mitochondrial dysfunction pathways in *Candida albicans*. RSC Adv..

[59] Mayer F.L., Wilson D., Hube B. (2013). Candida albicans pathogenicity mechanisms. Virulence.

[60] Flieger M., Bandouchova H., Cerny J., Chudickova M., Kolarik M., Kovacova V., Martinkova N., Novak P., Sebesta O., Stodulkova E. (2016). Vitamin B2 as a virulence factor in *Pseudogymnoascus destructans* skin infection. Sci. Rep..

