# Supplementary material for: Transcriptomic and Metabolomic Insights into the Inhibitory Mechanisms of Bat Cave Soil Microbial Volatiles Against Pseudogymnoascus destructans
Source: Microorganisms. 2026 Jul 6;14(7):1478. doi: 10.3390/microorganisms14071478 (PMC13413975; doi:10.3390/microorganisms14071478)
Supplement: Supplementary file 1 [file microorganisms-14-01478-s001.zip › microorganisms-4349518-supplementary.pdf]

Transcriptomic and Metabolomic Insights into the Inhibitory Mechanisms of Bat Cave Soil Microbial Volatiles Against *Pseudogymnoascus destructans*

Zihao Huang [1,†], Mingqi Shan [1,†], Shaopeng Sun [1], Denghui Wang [1], Fan Wang [1], Keping Sun [2], Zhongle Li [1,3,4,*] and Jiang Feng [1,2,3,4,*]

[1] College of Life Science, Jilin Agricultural University, Changchun 130118, China
[2] Key Laboratory of Vegetation Ecology of Education Ministry, Institute of Grassland Science, Northeast Normal University, Changchun 130024, China
[3] Jilin Provincial International Cooperation Key Laboratory for Biological Control of Agricultural Pests, Jilin Agricultural University, Changchun 130118, China
[4] Jilin Provincial Key Laboratory of Animal Resource and Ecological Security, Jilin Agricultural University, Changchun 130117, China
* Correspondence: lzy1514316@126.com (Z.L.); fengj@nenu.edu.cn (J.F.)
† These authors contributed equally to this work.

Figure S1. RT-qPCR validation of RNA-seq data.

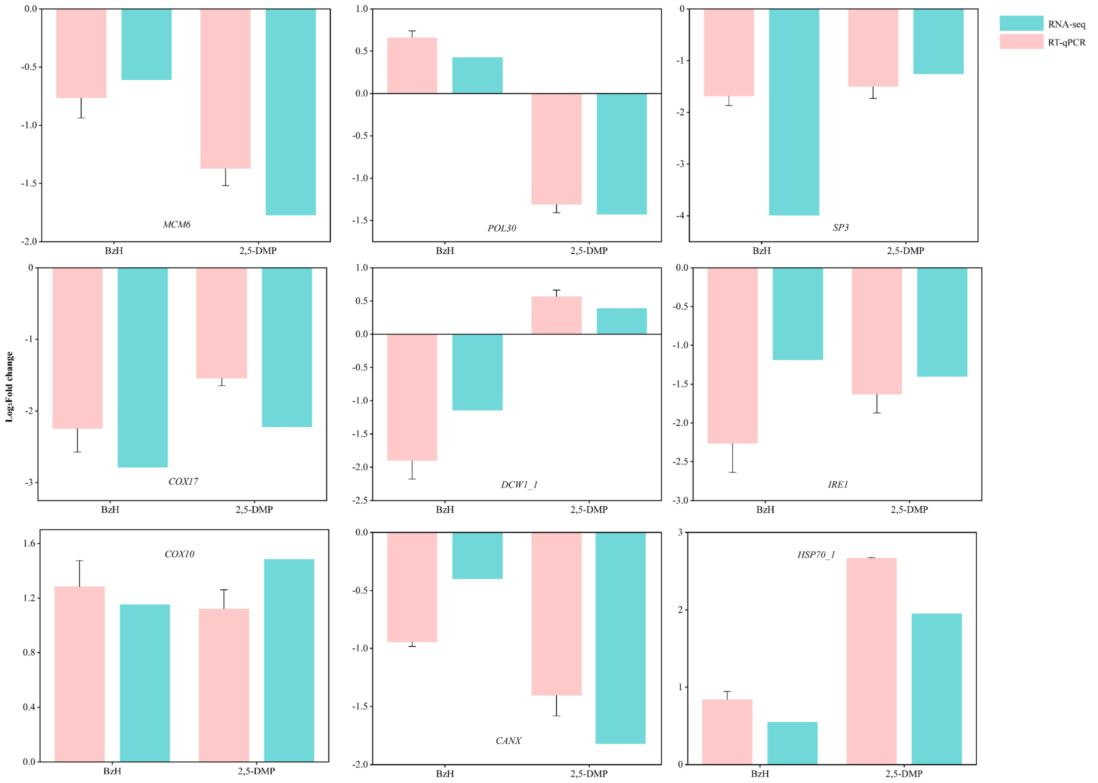

Table S1. Primers used for RT-qPCR.

Gene	Primers (5′-3′)	MW	GC Content (%)	TM (°C)	Transcript ID
DCW1_1	F: GCGCTTGAAGTGATCCAGAG	6183	55	59.9	XM_024465046.1
(VC83_01650)	R: ACGAGTCCCGCAAGAATCAC	6081	55	59.9	
IRE1	F: GGGAAGTTGTATGCCCGAGA	6223	55	59.9	XM_024472539.1
(VC83_09017)	R: TGACCAATCCTGGCTGAGGG	6159	60	61.9	
MCM6	F: CTTGCCCAAGAGGCTGTCC	5766	63.2	61.9	XM_024469125.1
(VC83_05572)	R: TGGATTTGCTCACACCAGACA	6391	47.6	58	
COX10	F: TCATAAGGCCGCTCTCACTC	6014	55	59.9	XM_024467234.1
(VC83_03325)	R: TTGAGACAATGTGCCGAGGA	6207	50	57.8	
SP3	F: GCGCGACAGACAGAAATGAC	6207	55	59.9	XM_024472612.1
(VC83_09074)	R: GCTGGTTCCGTTCTTCTCCA	6170	55	59.9	
CANX	F: CAAGTTCGTCCCCGAGGTT	5781	57.9	59.7	XM_024471593.1
(VC83_08030)	R: CTTGTTAACCTCTGCCTTCCC	6260	52.4	60	
POL30	F: ACCGACTACTCTGCTACTGTCT	6622	50	60.1	XM_024471592.1
(VC83_09664)	R: TCTGGCTTGTCGACGTTTGT	6122	50	57.8	
COX17	F: TCTTCAGCATCAATCCAGCTTT	6621	40.9	56.3	XM_024467802.1
(VC83_04361)	R:CCATGCAGCTCTTGTACTTGTC	6653	50	60.1	
HSP70_1	F: TCCAGGCTGCCATTCTATCCG	6334	57.1	61.9	XM_024464719.1
(VC83_01046)	R:TTGTCGGAGAAGGTGGAGAAC	6576	52.5	60	
EFG1	F:GATGGAGGAAGGCACACTGAA	6554	52.4	60	XM_024468911.1
(VC83_05289)	R: TCCGGAACACGTTCCATCT	6725	52.6	57.6	

F, forward primer; R, reverse primer. *EFG1*, reference gene

Table S2. Taxonomic identification and antifungal activity of the top 10 microbial strains.

Sampling Sites	Strain ID	Species Name	Inhibition Rate (%)	Sequence Identity (%)
Temple cave	TC1011	*Pseudomonas resinovorans*	100	100
Di cave	DC0321	*Pseudomonas putida*	100	100
Gezi Cave	GC0607	*Bacillus cereus*	100	100
Gezi cave	GC0115	*Stenotrophomonas rhizophila*	100	100
Temple cave	TC0213	*Acinetobacter rhizosphaerae*	97.42±1.5	100
Di cave	DC0206	*Pseudomonas arcuscaelestis*	96.34±2.8	100
Dicave	DC0816	*Pseudomonas yamanorum*	95.23±1.9	100
Di cave	DC0421	*Bacillus toyonensis*	93.62±2.2	100
Di cave	DC0509	*Pseudomonas wayambapalatensis*	91.54±2.1	100
Di cave	DC0916	*Pseudomonas sichumanensis*	90.51±3.1	100

Table S3. GC-MS identification of the top five most abundant volatile organic compounds produced by the four highly antagonistic bacterial strains after blank-control comparison.

Strain	Compound Name	CAS	Molecular Formula	RT(min)	Match Factor	Peak Area	Relative Abundance (%)	Blank Control
Pseudomonas resinovorans	2,5-dimethylpyrazine	123-32-0	C6H8N2	17.6983	96.79	11825151.19	13.05	No
	Alpha-Thujone	546-80-5	C10H16O	12.3327	94.20	9503394.55	10.49	No
	Hexadecamethylcyclooctasiloxane	556-68-3	C16H48O8Si8	19.7163	93.55	5087096.10	5.62	No
	methyl 2-methylprop-2-enoate	80-62-6	C5H8O2	4.9892	97.48	4583188.21	5.06	No
	Tetradecamethylcycloheptasiloxane	107-50-6	C14H42O7Si7	9.0318	80.46	3243522.51	3.58	No
Pseudomonas pudita	benzaldehyde	100-52-7	C7H6O	9.8736	98.37	30491140.51	25.46	No
	Tetradecamethylcycloheptasiloxane	107-50-6	C14H42O7Si7	17.6982	96.61	11423011.52	9.54	No
	Alpha-Thujone	546-80-5	C10H16O	12.3259	97.05	7258347.70	6.06	No
	Octamethylcyclotetrasiloxane	556-67-2	C8H24O4Si4	10.1342	95.98	7154319.77	5.97	No
	2-Methylpyrazine	109-08-0	C5H6N2	7.2476	96.22	5026680.19	4.20	No
Bacillus cereus	1-Eicosanol	629-96-9	C20H42O	16.4753	82.80	14100077.80	14.57	No
	Alpha-Thujone	546-80-5	C10H16O	12.3191	96.52	11289319.19	11.67	No
	Hexamethylcyclotrisiloxane	541-05-9	C6H18O3Si3	6.6328	98.99	6680536.61	6.90	No
	Tetradecamethylcycloheptasiloxane	107-50-6	C14H42O7Si7	17.6914	96.16	6146980.97	6.35	No
	Octamethylcyclotetrasiloxane	556-67-2	C8H24O4Si4	10.1341	96.73	5083471.07	5.25	No
Stenotrophomon	Benzaldehyde	100-52-7	C7H6O	9.8201	98.97	91467571.04	30.78	No
	Tetradecamethylcycl	107-50-6	C14H42O	17.6981	97.09	22023124.28	7.41	No

as rhizophila	oheptasiloxane		7Si7					
	Hexadecamethylcyclooctasiloxane	556-68-3	C16H48O8Si8	19.7161	93.56	13171231.45	4.43	No
	Dodecamethylcyclohexasiloxane	540-97-6	C12H36O6Si6	15.4196	95.81	11390156.71	3.83	No
	2-Methylundecane	7045-71-8	C12H26	13.1210	97.20	11220336.93	3.78	No

Table S4. Overview of RNA-seq read quality.

Sample	Raw Reads	Clean Reads	N Ratio(%)	Q30 Ratio(%)	GC Ratio(%)	Total Map
CK-1	42955696	41924824	0.01%	95.64%	55.35%	77.04%
CK-2	46734450	45440468	0.01%	95.52%	55.64%	75.84%
CK-3	47965170	46771954	0.01%	95.68%	55.68%	76.49%
CK-4	49325112	48066448	0.01%	96.42%	55.6%	76.9%
BzH-1	49320076	48027488	0.01%	95.71%	55.2%	90.06%
BzH-2	47662330	46418828	0.01%	95.83%	55.08%	88.75%
BzH-3	48033280	46560384	0.01%	95.58%	55.19%	89.47%
BzH-4	46975950	45689376	0.01%	95.71%	55.16%	88.11%
2,5-DMP-1	46124024	44635798	0.01%	95.95%	55.93%	89.62%
2,5-DMP-2	46600402	45475022	0.01%	95.49%	56.02%	90.42%
2,5-DMP-3	42972942	41563226	0.01%	95.43%	55.97%	89.05%
2,5-DMP-4	47436162	45971356	0.01%	95.94%	55.93%	88.67%

Table S5. Top 5 enriched GO terms in each primary category for DEGs following BzH and 2,5-DMP treatments.

Category	GO ID	Description	Gene Ratio	*P*-value	Up	Dwon
BP	GO:0055114	oxidation-reduction process	81/326	1.41E-05	21	60
BP	GO:0055085	transmembrane transport	57/326	0.015537741	17	40
BP	GO:0007049	cell cycle	8/326	0.036731232	1	7
BP	GO:0030001	metal ion transport	10/326	0.06205665	3	7
BP	GO:0007017	microtubule-based process	5/326	0.079301596	1	4
MF	GO:0016491	oxidoreductase activity	80/451	8.93E-06	20	60
MF	GO:0048037	cofactor binding	54/451	0.000122052	13	41
MF	GO:0016705	oxidoreductase activity, acting on paired donors, with incorporation or reduction of molecular oxygen	16/451	0.001875077	2	14
MF	GO:0005506	iron ion binding	18/451	0.002366534	2	16
MF	GO:0020037	heme binding	18/451	0.002366534	2	16
CC	GO:0016020	membrane	83/141	0.007275222	28	55
CC	GO:0016021	integral component of membrane	52/141	0.009643627	16	36
CC	GO:0031224	intrinsic component of membrane	52/141	0.009643627	16	36
CC	GO:0005886	plasma membrane	4/141	0.05733237	2	2
CC	GO:0015630	microtubule cytoskeleton	4/141	0.099137304	0	4

Category	GO ID	Description	Gene Ratio	*P*-value	Up	Dwon
BP	GO:0006259	DNA metabolic process	41/626	0.002212178	8	33
BP	GO:0022613	ribonucleoprotein complex biogenesis	17/626	0.00300952	0	17
BP	GO:0006260	DNA replication	18/626	0.004005231	2	16
BP	GO:0006364	rRNA processing	11/626	0.004235973	0	11
BP	GO:0016072	rRNA metabolic process	11/626	0.004235973	0	11
MF	GO:0020037	heme binding	28/864	0.001843704	17	11
MF	GO:0046906	tetrapyrrole binding	28/864	0.001843704	17	11
MF	GO:0048037	cofactor binding	79/864	0.010997721	46	33
MF	GO:0051536	iron-sulfur cluster binding	11/864	0.014947437	7	4
MF	GO:0051540	metal cluster binding	11/864	0.014947437	7	4

CC	GO:0016021	integral component of membrane	103/302	0.004522617	41	62
CC	GO:0031224	intrinsic component of membrane	103/302	0.004522617	41	62
CC	GO:0005783	endoplasmic reticulum	16/302	0.020799266	0	16
CC	GO:0000775	chromosome, centromeric region	10/302	0.022388251	1	9
CC	GO:1902494	catalytic complex	29/302	0.023366627	7	22

Table S6. Significantly enriched KEGG pathways of DEGs in the BzH and 2,5-DMP treatment groups.

**Pathway ID**	**KEGG-A-class**	**Pathway**	**Out**	**All**	***P*-value**
ko01120	Metabolism	Microbial metabolism in diverse environments	41	177	1.77E-05
ko01100	Metabolism	Metabolic pathways	130	815	2.86E-05
ko00620	Metabolism	Pyruvate metabolism	11	37	0.003607785
ko04122	Genetic Information Processing	Sulfur relay system	5	10	0.004085191
ko00260	Metabolism	Glycine, serine and threonine metabolism	10	36	0.009226529
ko01200	Metabolism	Carbon metabolism	20	95	0.009477841
ko00770	Metabolism	Pantothenate and CoA biosynthesis	7	22	0.01319914
ko00500	Metabolism	Starch and sucrose metabolism	10	38	0.01372614
ko00980	Metabolism	Metabolism of xenobiotics by cytochrome P450	5	13	0.01525573
ko00626	Metabolism	Naphthalene degradation	4	10	0.02590425
ko00621	Metabolism	Dioxin degradation	3	6	0.02787998
ko00624	Metabolism	Polycyclic aromatic hydrocarbon degradation	3	6	0.02787998
ko00950	Metabolism	Isoquinoline alkaloid biosynthesis	3	6	0.02787998
ko00040	Metabolism	Pentose and glucuronate interconversions	6	20	0.02865854
ko00361	Metabolism	Chlorocyclohexane and chlorobenzene degradation	2	3	0.04175332
ko00590	Metabolism	Arachidonic acid metabolism	2	3	0.04175332
ko00623	Metabolism	Toluene degradation	2	3	0.04175332
ko00350	Metabolism	Tyrosine metabolism	5	17	0.04857623
ko00460	Metabolism	Cyanoamino acid metabolism	5	17	0.04857623

**Pathway ID**	**KEGG-A-class**	**Pathway**	**Out**	**All**	***P*-value**
ko00910	Metabolism	Nitrogen metabolism	13	16	1.97E-05

ko04714	Organismal Systems	Thermogenesis	33	69	0.000539369
ko04141	Genetic Information Processing	Protein processing in endoplasmic reticulum	34	74	0.001106847
ko03030	Genetic Information Processing	DNA replication	18	34	0.002518466
ko00190	Metabolism	Oxidative phosphorylation	32	76	0.008197818
ko00920	Metabolism	Sulfur metabolism	8	13	0.01406804
ko02020	Environmental Information Processing	Two-component system	12	24	0.02270367
ko04913	Organismal Systems	Ovarian steroidogenesis	3	3	0.02399543
ko04612	Organismal Systems	Antigen processing and presentation	5	7	0.02417972
ko01120	Metabolism	Microbial metabolism in diverse environments	63	177	0.02531544
ko03008	Genetic Information Processing	Ribosome biogenesis in eukaryotes	28	70	0.02763887
ko00982	Metabolism	Drug metabolism - cytochrome P450	7	12	0.03110059
ko04212	Organismal Systems	Longevity regulating pathway - worm	11	23	0.04123762
ko00430	Metabolism	Taurine and hypotaurine metabolism	5	8	0.04933226

Table S7. Significantly enriched KEGG pathways of shared DEGs in *P. destructans* under BzH and 2,5-DMP treatments.

Pathway ID	KEGG-A-class	Pathway	Out	All	*P*-value
ko01120	Metabolism	Microbial metabolism in diverse environments	24	177	0.000395867
ko01100	Metabolism	Metabolic pathways	72	815	0.000915786
ko00620	Metabolism	Pyruvate metabolism	8	37	0.002368621
ko00361	Metabolism	Chlorocyclohexane and chlorobenzene degradation	2	3	0.01270858
ko00590	Metabolism	Arachidonic acid metabolism	2	3	0.01270858
ko04122	Genetic Information Processing	Sulfur relay system	3	10	0.02470692
ko00720	Metabolism	Other carbon fixation pathways	3	11	0.03234356
ko00982	Metabolism	Drug metabolism - cytochrome P450	3	12	0.04106464

Table S8. Top 15 enriched KEGG pathways of DEMs in the BzH and 2,5-DMP treatment groups.

Pathway ID	KEGG-A-class	Pathway	Out	All	*P*-value
ko00380	Metabolism	Tryptophan metabolism	16	20	9.59E-05
ko00999	Metabolism	Biosynthesis of various plant secondary metabolites	12	18	0.01042049
ko04978	Organismal Systems	Mineral absorption	6	7	0.01285676

ko01100	Metabolism	Metabolic pathways	111	269	0.02517542
ko00996	Metabolism	Biosynthesis of various alkaloids	6	8	0.03535524
ko00440	Metabolism	Phosphonate and phosphinate metabolism	3	3	0.05273251
ko04726	Organismal Systems	Serotonergic synapse	4	5	0.06901038
ko00030	Metabolism	Pentose phosphate pathway	5	7	0.07354347
ko00121	Metabolism	Secondary bile acid biosynthesis	5	7	0.07354347
ko00966	Metabolism	Glucosinolate biosynthesis	5	7	0.07354347
ko00360	Metabolism	Phenylalanine metabolism	6	10	0.1268393
ko04122	Genetic Information Processing	Sulfur relay system	2	2	0.1411871
ko04361	Organismal Systems	Axon regeneration	2	2	0.1411871
ko00052	Metabolism	Galactose metabolism	3	4	0.1520836
ko00460	Metabolism	Cyanoamino acid metabolism	3	4	0.1520836

Pathway ID	KEGG-A-class	Pathway	Out	All	*P*-value
ko01100	Metabolism	Metabolic pathways	127	269	0.007624089
ko00999	Metabolism	Biosynthesis of various plant secondary metabolites	13	18	0.009582376
ko00030	Metabolism	Pentose phosphate pathway	6	7	0.02585644
ko00380	Metabolism	Tryptophan metabolism	13	20	0.03376254
ko00970	Genetic Information Processing	Aminoacyl-tRNA biosynthesis	8	11	0.04198842
ko04974	Organismal Systems	Protein digestion and absorption	10	15	0.05047795
ko00760	Metabolism	Nicotinate and nicotinamide metabolism	7	10	0.07503721
ko00051	Metabolism	Fructose and mannose metabolism	3	3	0.07702442
ko01230	Metabolism	Biosynthesis of amino acids	21	39	0.09562978
ko04726	Organismal Systems	Serotonergic synapse	4	5	0.1079244
ko00710	Metabolism	Carbon fixation by Calvin cycle	5	7	0.1226219
ko00966	Metabolism	Glucosinolate biosynthesis	5	7	0.1226219
ko04978	Organismal Systems	Mineral absorption	5	7	0.1226219
ko00130	Metabolism	Ubiquinone and other terpenoid-quinone biosynthesis	6	9	0.1297791
ko00270	Metabolism	Cysteine and methionine metabolism	7	11	0.1328726

Table S9. List of putative virulence factors.

Gene	log2FC (BzH/2,5-DMP)	FDR (BzH/2,5-DMP)	Full Name	Blastx
**Secreted proteases**				
VC83_09074	-3.994/-1.260	0.056/0.008	Subtilisin-like protease 3	-
VC83_06060	-2.051/-	6.03978E-21/-	Probable aspartic-type endopeptidase OPSB	OPSB_ARTBC
VC83_00241	-2.442/-2.537	8.52076E-72/1.96664E-19	Aspergillopepsin-2	PRTA_ASPNG
VC83_00947	-/1.465	-/9.49733E-31	Lipase	LIP_THELA
VC83_01361	-/1.744	-/2.6056E-08	Major allergen Asp f 2	ALL2_ASPFU
VC83_01226	-1.090/-	7.68498E-44/-	Secreted lipase ARB07186/07185	LIP1_ARTBC
**Cell Wall Remodeling**				
VC83_03500	-3.076/-2.111	1.08792E-84/4.06589E-14	Spherulin-1A	SR1A_PHYPO
VC83_07867	-/1.929	-/2.65658E-22	Uncharacterized protein AFUA_6G02800	YA280_ASPFU
VC83_07327	-1.725/-	1.25326E-40/-	Probable glucan endo-1,3-beta-glucosidase eglC	EGLC_NEOF
VC83_02115	-/-1.235	-/2.90986E-14	hydrolase 76 protein	-
VC83_07145	-/-1.162	-/5.74759E-06	hydrolase 76 protein	-
VC83_01650	-1.146/-	7.84355E-25/-	hydrolase 76 protein	-
VC83_09076	-1.341/-	6.60875E-21/-	Probable glucan endo-1,3-beta-glucosidase ARB_02077	E13B_ARTBC
VC83_08448	-2.574/-1.520	1.0904E-136/3.7658E-21	Protein SUR7	SUR7_CANAL
VC83_01650	-1.146/-	7.84355E-25/-	Mannan endo-1,6-alpha-mannosidase DCW1	DCW1_YEAST
VC83_06199	-/-1.820982164	-/1.22754E-25	Chitin synthase 2	-
VC83_01007	-2.388/-	0.001/-	Probable glucan 1,3-beta-glucosidase D	EXGD_NEOF
**Ion Homeostasis**				
VC83_01360	-1.451/-	8.21492E-13/-	Zinc-regulated transporter 1	ZRT1_SCHPO
VC83_07026	-/2.418	-/6.61044E-20	Na+ ATPase	-
VC83_06862	-/1.082	-/1.66087E-06	Calcium-transporting ATPase 3	ATC3_SCHPO
VC83_01014	-1.131/-	4.69115E-51/-	Calcium-transporting ATPase 2	ATC2_SCHPO
VC83_04094	-1.223/-2.031	1.45789E-28/5.58423E-57	Cytosolic copper metallochaperone	-
VC83_07127	-/2.755	-/3.98951E-21	Na+ ATPase	-
VC83_01033	1.421/1.633	1.67295E-37/5.76723E-20	Zinc-regulated transporter 2	ZRT2_YEAST
VC83_00415	-1.347/-2.823	6.93372E-17/1.9231E-136	low-affinity Fe(2+) transport protein	-
**Heat shock response**				

VC83_02553	1.348/2.893	0.0003/3.1205E-75	30 kDa heat shock protein	HSP30_NEUCR
VC83_08137	1.415/3.017	4.74964E-36/1.35382E-34	Heat shock protein hsp98	-
VC83_00970	2.093/3.341	1.5599E-35/1.6644E-139	chaperone ATPase hsp78	-
VC83_01046	-/1.955	-/1.20504E-21	Hsp70 chaperone	-
VC83_08187	-/1.406	-/2.23966E-24	Heat shock protein 9	-
VC83_01964	-/2.879	-/5.43243E-79	adenyl-nucleotide exchange factor sse1	-
VC83_06435	-/1.137	-/1.49209E-19	Hsp90 cochaperone	-
VC83_00686	-/1.061	-/1.33381E-37	Heat shock protein 60, mitochondrial	HSP60_PARBA
VC83_04853	-/1.542	-/4.38191E-30	Type I HSP40 co-chaperone	-
VC83_07843	-/2.492	-/3.51443E-19	hsp70 nucleotide exchange factor fes1	-
VC83_06414	-/-1.414	-/6.60755E-34	Heat shock protein ssb1	-
